# Evaluation of Corneal Endothelium in Children and Adolescents with Type 1 Diabetes Mellitus

**DOI:** 10.1155/2013/913754

**Published:** 2013-12-08

**Authors:** Beata Urban, Dorota Raczyńska, Alina Bakunowicz-Łazarczyk, Krystyna Raczyńska, Małgorzata Krętowska

**Affiliations:** ^1^Department of Pediatric Ophthalmology and Strabismus, Medical University of Bialystok, Waszyngtona Street 17, 15-274 Białystok, Poland; ^2^Department of Ophthalmology, Medical University, Mariana Smoluchowskiego 17, 80-214 Gdańsk, Poland; ^3^Bialystok University of Technology, 45A Wiejska Street, 15-351 Białystok, Poland

## Abstract

*Purpose*. To evaluate the systemic and local factors that contribute to the damage of endothelial cells in diabetic patients and to compare the endothelial structure of the cornea in diabetic and nondiabetic patients. *Materials and Methods*. The endothelial cell density (ECD) and central corneal thickness (CCT) were investigated in 123 eyes of type 1 diabetic patients and in 124 eyes of nondiabetic patients. The mean diabetic patients age was 15.34 ± 3.06 years versus 14.58 ± 2.01 years in the control group. The mean duration of diabetes was 8.02 ± 3.9 years. The corneal endothelium was imaged by the Topcon SP-2000P. *Results*. The mean ECD in diabetic eyes was 2435.55 ± 443.43 cells/mm^2^ and was significantly lower than in control group (2970.75 ± 270.1 cells/mm^2^). The mean CCT was 0.55 ± 0.03 mm in diabetic group versus 0.53 ± 0.033 mm in control group. ECD and CCT significantly correlated only with duration of diabetes. There was no correlation between ECD and CCT and patient age, sex, HbA1C level, and plasma creatinine level. *Conclusions*. ECD is decreased and CCT is increased in children and adolescents with diabetes mellitus. Duration of diabetes is the factor that affects ECD and CCT.

## 1. Introduction

The corneal endothelium is a single layer of uniformly sized cells with hexagonal shape. Their amount decreases by approximately 0.5%-0.6% (100–200 cells) per year [1]. The endothelial cell dysfunction is observed in myopia and in contact lens wearers [2, 3]. The decreasing number of endothelial cells can also be a result of a surgical injury related to the opening of the anterior chamber [4]. Many studies have shown that even minor changes in the morphology of the endothelial cells may manifest in the disturbances in the tightness of the endothelial barrier. It has been demonstrated that human corneal endothelial cells have mitotic ability *in vitro*, but *in vivo* they do not exit the cell cycle but are arrested in G1 phase [5]. Loss of cells is compensated through the expanding and spreading of cells, which over time results in a lack of tightness and corneal oedema. Prevention of the corneal endothelium dysfunction, its early detection and immediate treatment are therefore crucial, especially if the problem concerns young patients. Noncontact specular microscopy, which evaluates endothelial morphology quickly and easily, can be especially useful with children.

One of the conditions, which affect the cornea is diabetes. There are many reports concerning the analysis of the corneal endothelium in adults with type 1 and type 2 diabetes [6–9]; however, there are no publications concerning the analysis of the cornea in juvenile patients with this disease. The aim of our study was to compare the endothelial cell density and central corneal thickness in diabetic and nondiabetic patients and to evaluate the local and systemic factors which may affect the corneal endothelium in this group.

## 2. Materials and Methods

The current study was performed at the Department of Pediatric Ophthalmology and Strabismus, Medical University of Bialystok, Poland. This investigation received approval from the University Ethic Committee. For the purpose of this study  we examined 123 eyes of 123 patients with type 1 diabetes (60 boys and 63 girls). The age of diabetic group was 7–19 years (mean: 15.34 ± 3.06 years). The mean duration of diabetes was 8.02 ± 3.9 years and ranged from 8 months to 16 years. All the diabetic patients were divided into three groups according to diabetes duration: less than 5 years (38 patients), from 5 to 10 years (42 patients), and longer than 10 years (43 patients). 48 persons had bad metabolic control, 37 had moderate metabolic control, and 38 had good metabolic control. At the time of examination, the mean value of HbA1c in diabetic patients was 8.02 ± 3.9% (range 5.5%–3.2%). Ophthalmologic examination in diabetic patients included slit-lamp examination and binocular indirect ophthalmoscopy fundus examination.

As controls, 124 eyes of 124 patients (66 boys and 58 girls) were examined. The mean age of the control group was 9–18 years (mean: 14.58 ± 2.01 years). None of the examined patients had history of ocular disease, topical ocular medications, or contact lens wear.

Data from the right eye of each patient was used in this study. The corneal endothelium density (ECD) and central corneal thickness (CCT) in its central part were diagnosed using the Topcon SP-2000P endothelial microscope. Several pictures were taken until a clear image of the endothelium was obtained. The endothelial cell count was performed using built-in image analysis software. On clear image 25 cells were counted manually. CCT was measured automatically. The image with the analyzed data was then printed out.

The aim of this study was to compare ECD and CCT in diabetic and nondiabetic patients and to evaluate a correlation between endothelial cell density, central corneal thickness, and local factors (presence of retinopathy) and systemic factors (age, sex, diabetes duration, the level of HbA1c, and plasma creatinine level).

### 2.1. Statistical Analysis

The Mann-Whitney test for ECD and *t*-test for CCT were used to compare medians in diabetic and control group. The Kruskal-Wallis test and ANOVA test were used to compare the values of medians in different states of metabolic control. Multiple regression analysis was used to analyze the influence of the set of variables for ECD and CCT. In the model four continuous variables (age, diabetes duration, plasma creatinine level, and HbA1c) and two dummy variables (sex and presence of retinopathy) were used. In our study the starting model and the final model received by the use of backward elimination of variables are presented. The value of multiple R, R2, and *P* value for the global test that R2 is equal to 0. The normal distribution of the residuals was verified by the use of Pearson's chi square test. The analysis was performed using the PRISM packet. Differences with *P* value less than 0.05 were considered statistically significant.

## 3. Results

**Figure 1:**
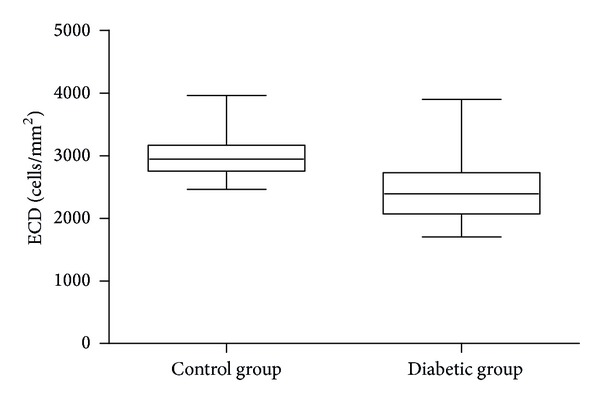
ECD values in patients with diabetes and in the control group.

A group of 123 children and adolescents with type 1 diabetes were examined. The mean duration of diabetes was 8.02 ± 3.9 years. The mean endothelial cell density in patients with diabetes was 2435.55 ± 443.43 cells/mm² and was significantly lower than in the control group (2970.75 ± 270.1 cells/mm²) (*P* = 0.0001; Mann-Whitney test). ECD values in both groups are presented in Figure 1

**Figure 2:**
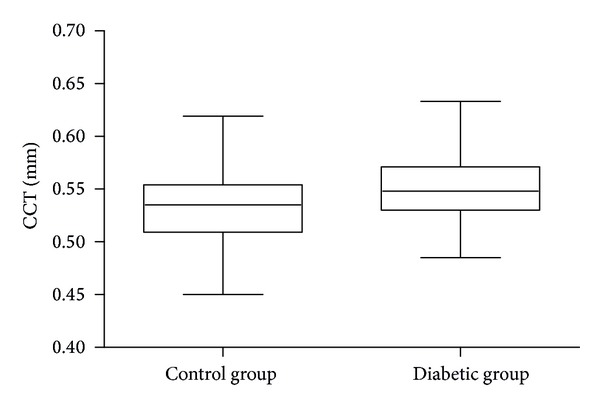
CCT values in patients with diabetes and in the control group.

The mean CCT was 0.55 ± 0.03 mm in diabetic group versus 0.53 ± 0.033 mm in control group (*P* < 0.0001; *t*-test). CCT values in both groups are presented in Figure 2

In order to determine the systemic and local factors affecting the corneal endothelium in diabetic patients we evaluated the correlation of ECD and CCT with the following variables: the patients' age and sex, duration of diabetes, HbA1c level, plasma creatinine level, and presence of diabetic retinopathy.

The age of diabetic group was 7–19 years (mean: 15.34 ± 3.06 years). There was no correlation between ECD and age of diabetic patients (*P* value = 0.111). The mean ECD was 2446 ± 488.3 cells/mm² in diabetic boys and 2424 ± 394.7 cells/mm² in diabetic girls, and these differences were not statistically significant (*P* value = 0.99). 38 diabetic patients had good metabolic control (with HbA1c less than 7%), 37 subjects had moderate metabolic control (HbA1c from 7% to 8%), and 48 persons had poor metabolic control (HbA1c above 8%). We did not observed significant differences in ECD in relation to metabolic control (*P* value = 0.54). The mean duration of diabetes was 8.02 ± 3.9 years (ranged from 8 months to 16 years). In 38 patients duration of diabetes was shorter than 5 years, 42 persons suffered from diabetes from 5 to 10 years, and 43 persons had diabetes longer than 10 years. These differences were statistically significant (*P* value = 0.001). The mean ECD values in relation to sex, metabolic control, and duration of diabetes are presented in Table 1.

**Figure 3:**
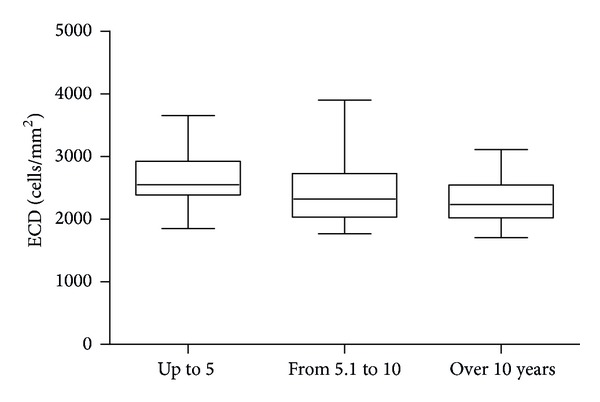
ECD values in relation to the duration of diabetes.

ECD values in relation to the duration of diabetes are presented in Figure 3.

The mean HbA1c in diabetic patients was 8.26 ± 1.7% and ranged from 5.5% to 13.2%. There was no significant correlation between ECD and HbA1c level (*P* value = 0.378). There was no correlation between ECD and plasma creatinine level (*P* value = 0.650). We did not observe any correlation between ECD and the presence of diabetic retinopathy (*P* value = 0.293). Only 5 patients had nonproliferative diabetic retinopathy (their ECD was 2045.25 ± 240.46 cells/mm² versus 2452.89 ± 442.75 cells/mm² in the remaining patients without retinopathy). The correlation between ECD and duration of diabetes was statistically significant (*P* value <0.0001) (Table 2).

The mean CCT value in patients with duration of diabetes up to 5 years was 0.539 ± 0.027 mm, with duration of diabetes from 5 to 10 years was 0.551 ± 0.03 mm, and with duration of diabetes over 10 years was 0.558 ± 0.03 mm, and the differences were statistically significant (*P* value = 0.0144; ANOVA test).

Multiple regression analysis for CCT indicated that only duration of diabetes was significantly related to CCT. The correlations between CCT and systemic and local factors are presented in Table 3.

## 4. Discussion

Corneal changes are diagnosed in about 70% of adult patients with diabetes [6]. The observed disorders include increased fragility and damage of the corneal endothelium and recurring erosions and increased sensitivity to injuries [7]. Experimental research discovered abnormal basement membrane of the endothelium, a decreased number of hemidesmosomes, and a prolonged healing of the cornea and its decreased sensitivity [7]. Many studies confirmed that diabetes causes abnormalities in morphology and functioning of corneal endothelium cells. Functional disturbances may lead to increased autofluorescence of the cornea and its increased penetrability [8, 9]. Morphological changes may result in a high variability factor of the endothelial cell surface and decreased percentage of hexagonal cells in corneas in patients with diabetes, using contact specular microscope, when compared to healthy patients [8–11]. Although morphology of the endothelial cells is interesting in diabetic patients, the limitations of Topcon 2000 are obvious (pleomorphism and polymegathism could not be assessed, and hexagonality was also not available). The existing research data encouraged us to examine the corneas in children and adolescents with this chronic illness.

We have established that the mean density of corneal endothelium cells in patients with diabetes was reduced—in comparison with the control group—by 18%. Similar results were obtained by Roszkowska et al., who after examining 75 adults with type 1 and type 2 diabetes noted the ECD decreased by 5% in type 2 diabetes, and by 11% in type 1 when compared with healthy persons [10]. Lower endothelial cell counts were also proved by Sudhir et al., who examined 1191 adult patients with type 2 diabetes [12]. The mean ECD in their study was 2550 ± 326 cells/mm² versus 2634 ± 256 cells/mm² in the nondiabetic control group. In our diabetic group ECD was very similar (2435.55 ± 443.43 cells/mm²) but ECD in our control group was higher (2970.75 ± 270.1 cells/mm²). We have to remember that our diabetic and non-diabetic subjects were young: the mean age in diabetic group was 15.34 ± 3.06 years versus 14.58 ± 2.01 years in control group. Different observations were done by Furuse et al., who did not demonstrate significant changes in mean density of corneal endothelium cells in diabetic subjects, but they only examined patients with type 2 diabetes [13].

We also analyzed the influence of local and systemic factors affecting the density of the corneal endothelium in children and adolescents with type 1 diabetes. We demonstrated a significant correlation between ECD and only the duration of diabetes. In contrast, Inoue et al. did not find any ocular and systemic factors that affect the damaging of endothelium in diabetic patients, but it was type 2 diabetes [14]. The authors claim that although they did not show the influence of systemic and ophthalmic factors on the morphology of the corneal endothelium, due to the chronicity of the disease, the relation may not occur during the examination but it may appear some years later. Larsson et al. noticed several changes in the endothelium in elderly with diabetes, but the observed anomalies may have been additionally caused by the process of senescence [9]. Similarly to our study, Lee et al. showed that ECD in adult diabetic patients was significantly lower for diabetes with over 10 years of duration than for diabetes of under 10 years [15].

Like other authors, we did not prove the influence of sex, the state of metabolic control, and the presence of diabetic retinopathy on the corneal endothelium [11, 16, 17]. Ziadi et al. did not detect any relation between the level of glycosylated haemoglobin and the condition of the corneal endothelium [18]. The patients they have examined had similar values of HbA1c (mean 8.2%) to the mean HbA1c level of our patients (8.01%). They did not detect any influence of diabetes duration (in contrast to our study) and the presence of diabetic retinopathy (similarly to our results) on the corneal endothelium. In the study of Módis et al., the HbA1c level in adult patients with type 1 diabetes mellitus was inversely correlated with the ECD, but they did not prove such correlation in patients with type 2 diabetes mellitus [19]. The authors concluded that type 1 diabetic corneas are more susceptible to environmental changes than type 2 diabetic corneas.

We were unable to demonstrate any correlation between ECD and the presence of diabetic retinopathy. Siribunkum et al. while examining 64 adult patients with diabetes mellitus observed that the severity of diabetic retinopathy was correlated with endothelial cell density, but these correlations were low and the corneal changes were not correlated with glycemic control [17]. Inoue et al. reported that the presence of retinopathy (proliferative also) as well as laser coagulation in medical history did not affect the density of corneal endothelium cells [14]. Recently, the role of growth factors, CRP, proinflammatory cytokines, or level of lipids has been emphasised in the pathogenesis of diabetic retinopathy in children [20, 21]. Their significantly higher blood serum levels have noxious influence on the tiny blood vessels in the retina. Specificity of the cornea—first of all lack of blood vessels—may be one of the reasons, that in many publications concerning with the corneal endothelium in diabetic adults, the analysis of the influence of these factors was not performed [11, 14, 17–19, 22]. We did not check them either.

The research done by Roszkowska et al. shows that the corneal endothelium is a tissue undergoing constant metabolic stress [10]. In normal endothelial cells, circumferential bands of F-actin (a major component of the cellular cytoskeleton) help to maintain the regular and functionally efficient, hexagonal shape. Kim et al. demonstrated that the corneas of diabetic individuals showed marked irregular F-actin fibers crossing the endothelial cell cytoplasm [23]. They suggested, that these abnormal patterns of F-actin may be the result of constant stress in cell volume regulation in the corneas of diabetic patients. Kleinzeller and Ziyadeh showed that dissociation of F-actin fibers either chemically or osmotically caused massive cellular swelling [24]. They presumed that this abnormal collocation of F-actin in the endothelium of diabetics may contribute to altered morphology and, in their opinion, the mechanism may be related to sorbitol accumulation within these cells. Fujishima and Tsubota claim that in molecular pathogenesis of corneal changes significant importance may be given to aldose reductase, the first enzyme of the sorbitol pathway [25]. Aldose reductase has been demonstrated immunohistochemically in the corneal endothelium, and the osmotic stress that occurs secondary to sorbitol accumulation could lead to altered endothelial morphology and cell loss [26]. Ohguro et al. reported that alterations in endothelial morphology resolve within 3 months after the onset of topical aldose reductase inhibitor treatment [27].

Other probable reasons of changes in the endothelium include the glycation of membrane ATPases. The accumulation of advanced glycation end products (AGEs) in the epithelial basement membrane or in Descemet's membrane may play a role in the disorders of diabetic cornea [28]. Kaji et al. showed that AGEs formation on fibronectin and laminin attenuated the attachment and spreading of the corneal endothelial cells. They concluded, that AGEs formation in Descemet's membrane may be responsible for the corneal endothelial abnormalities in diabetic patients. The next mechanism could decrease Na^+^/K^+^-ATPase activity, which influences the endothelial pump action and induces the dysfunction of the corneal endothelial cell layer [18, 29]. Disturbances in this pump may lead to changes in corneal thickness. Thickness of the cornea indirectly informs about the functioning of the endothelial layer, which plays role as pump, which is responsible for active dehydration of the cornea and also has a barrier function. In our study we demonstrated that CCT was significantly thicker in diabetic group than in control group. This is in agreement with the findings of Roszkowska et al. but is in contrast with the work of Inoue et al. [10, 14]. We also observed significant correlation between duration of diabetes mellitus and CCT. Possible explanations for increased corneal thickness in diabetic patients include (besides inhibition of the corneal endothelial pump) an increased endothelial permeability, which result from the metabolic effects of diabetes. Another reason could be the increased stromal swelling pressure due to the accumulation of sorbitol or from the glycosylation of corneal collagen [30].

Evaluating the condition of the corneal endothelium is important since one of the most frequent reasons of corneal endothelium cells loss is cataract surgery, and cataract, besides diabetic retinopathy, is one of the most common ophthalmic complications of diabetes. The research done by Mathew et al. shows that the removal of cataract is exceptionally traumatizing for the endothelium in eyes of diabetic patients. The diabetic endothelium was found to be under greater metabolic stress and had less functional reserve after manual small incision cataract surgery [31]. It can be assumed that for children suffering from diabetes, an eventual development of cataract in the future and the necessity for its surgery may be a factor that significantly increases the risk of dysfunction of corneal endothelium cells. Shenoy concluded that evaluation of corneal endothelium in diabetic patients should be part of the protocol for eye care of diabetic patients [22].

## 5. Conclusions

The results of this study may support the theory of lower endothelial cell density and thicker cornea in children and adolescents with type 1 diabetes mellitus. Duration of diabetes is the factor that affects ECD and CCT and observed changes could predispose to corneal dysfunction in the future. Undoubtedly, further observations of greater numbers of young diabetic patients are essential.

## Figures and Tables

**Table 1:** Mean ECD values in relation to sex, metabolic control, and duration of diabetes.

Variables	ECD (mean ± stand dev.)	*P* value
Sex		0.99^a^
Male	2446 ± 488.3	
Female	2424 ± 394.7	
Metabolic control		0.54^b^
Good	2379 ± 385.3	
Moderate	2508 ± 497.2	
Poor	2424 ± 444.2	
Diabetes duration		0.001^b^
Up to 5 years	2653.842 ± 436.8	
From 5 to 10 years	2396.857 ± 454.8	
Over 10 years	2280.442 ± 363.2	

^a^ *P* value for Mann-Whitney test.

^b^ *P* value for Kruskal-Wallis test.

**Table 2:** Multiple regression analysis for ECD.

	Standardized regression coefficient *B*	Standard error of *B*	Regression coefficient *B*	*P* value
Intercept			2821.953	0.000

Age	−0.174	0.109	−25.265	0.111
Diabetes duration	−0.344	0.097	−39.150	0.001
Plasma creatinine level	0.050	0.109	164.937	0.650
HbA1c	0.077	0.086	23.911	0.378
Sex	0.032	0.085	28.559	0.703
Retinopathy	−0.094	0.089	−210.814	0.293

FINAL MODEL				

Intercept			2810.733	0.000

Diabetes duration	−0.411	0.083	−46.783	<0.0001

**Table 3:** Multiple regression analysis for CCT.

	Standardized regression coefficient *B*	Standard error of *B*	Regression coefficient *B*	*P* value
Intercept			0.524	0.000

Age	−0.048	0.115	0.000	0.680
Diabetes duration	0.293	0.103	0.002	0.005
Plasma creatinine level	−0.012	0.116	−0.003	0.919
HbA1c	0.083	0.092	0.002	0.366
Sex	0.079	0.090	0.005	0.380
Retinopathy	0.013	0.095	0.002	0.893

FINAL MODEL				

Intercept			0.532	0.000

Diabetes duration	0.288	0.087	0.002	0.001
